# Patients’ and Providers’ Preferences and Perceptions for Imaging Information for Patients: Cross-Sectional Survey Study

**DOI:** 10.2196/72362

**Published:** 2025-10-28

**Authors:** Eline M van den Broek-Altenburg, Nicholas OV Cunningham, Jamie S Benson, Naiim S Ali, Kristen K DeStigter

**Affiliations:** 1 Department of Radiology University of Vermont College of Medicine Burlington, VT United States; 2 Department of Radiology Boston Medical Center Boston, MA United States; 3 Perelman School of Medicine University of Pennsylvania Philadelphia, PA United States

**Keywords:** 21st Century Cures Act, results communication, imaging, decision-making, radiologists, patients, preferences, shared decision-making

## Abstract

**Background:**

Communication of imaging results is increasingly directed to patients, but controversies remain regarding report communication from the perspectives of patients, ordering providers, and radiologists.

**Objective:**

The objective of this study was to compare and contrast patients and providers with respect to their preferred source of imaging report information, preferred method of imaging report communication, and perceptions of patients’ understanding of imaging reports.

**Methods:**

We gathered preferences from patients and providers through surveys. In total, 91 patients as well as 77 physicians, 10 physician assistants, 6 nurse practitioners, and 1 other health provider completed the surveys. Chi-square and 2-tailed *t* tests were used to compare differences in means between the groups. Logistic regression was used to analyze the probability of an ordering provider preferring online release of imaging results as the first method of communication as a function of provider characteristics.

**Results:**

Of the 94 providers who participated in the study, 53 (56%) were women and 80 (85%) were White. On average, they had 15.6 (SD 10) years of experience. Most providers preferred delaying the release of imaging reports to patients until after they had reviewed the report themselves. There was substantial provider preference heterogeneity regarding imaging report communication to patients and the timing of release. The majority of the patients (70/91, 77%) who completed the survey were women, and 19% (17/91) identified as members of racial and ethnic minoritized groups. Patients generally preferred to receive their imaging results online as soon as they were available.

**Conclusions:**

The findings of this study suggest that shared decision-making between patients and providers before the release of imaging results could help establish how, when, and by whom results should be delivered to patients. The study findings can be leveraged to explore options for a differentiated reporting approach that is more responsive to patient and provider needs.

## Introduction

### Background

Radiology departments are increasingly directing communication of results to patients, but controversies remain regarding imaging report communication from the perspectives of patients, ordering providers, and radiologists. Imaging 3.0, the value-based care initiative of the American College of Radiology [1], dedicates a pillar to patient- and family-centered care, with information sharing with patients as a core component. This is consistent with the 21st Century Cures Act (hereinafter Cures Act) Final Rule, implemented in April 2021, which aims to put patients at the center of their health information by guaranteeing complete access to their medical imaging records, with limited exceptions [2]. For imaging reports, this means that the Cures Act mandates that imaging reports must immediately be made available to patients through an online portal. The implications of this law are widespread, affecting patient-provider relationships and almost every aspect of health care.

Disclosure of imaging reports has been investigated from several perspectives regarding how, when, and by whom results are delivered to patients even before the Cures Act was implemented [3-8]. In the United States, mandated direct communication of results to patients was first implemented under state law in Pennsylvania in October 2018, driven by concern over the increasing caseload faced by health care providers. The Patient Test Results Information Act (Pennsylvania Act 112) mandated that patients should be directly notified of a “significant abnormality” that would require follow-up care within three months and that this notification must occur within 20 days of the imaging examination [9]. Practices were not required to provide patients with a copy of the report or even the finding of concern, rather only that patients be made aware of the recommendations for follow-up.

Early analysis of the impact of Act 112 [10] suggested that it had had little effect on patient care.

### Preference Heterogeneity

Prior surveys of both patients and providers indicate that, while both groups favor more transparency in access to health information and imaging results, there are significant differences regarding how this information should be made available. Preference heterogeneity refers to the extent of variation among patients or providers in their preferences for direct reporting of results [11]. It can be influenced by factors such as the desired mix of attributes or the amount of each attribute preferred in goods and services [12].

General practitioners (GPs) have expressed concerns that patients may misinterpret their current symptoms by comparing them to their previous experiences or those of family members or friends [13]. One study [13] shared physicians’ comments:

Some struggling with persistent pain would ask for X-rays because they were familiar with these types of scans and know that it is available to them. You know they’ve put up with back pain so much they want to fix it quickly so they can get back to work.

GPs have also expressed worry that transparency would lead to induced demand [13]:

These individuals are increasingly obsessed with their health, they are particularly anxious and want reassurance with X-ray.

Conversely, patients believe that information should be made available to them directly, but they would still rely on physicians to make clinical decisions [14]. One study [14] reported comments from patients emphasizing that the physician’s expertise would still prevail:

The Internet is part of my life medically, but only a relatively small part. I rely mostly on my GP and specialist for most things.

Other patients in this study [14] reported similar views:

Yes, and [my doctor is] very good with latest research and things. He will say, “Oh I read this, and that doesn’t work anymore.” What was that thing that I was going to talk to him about next time that’s been recently in the TV—is it statins for the high blood pressure? That’s one thing I will ask him. So I’d rather trust him than say, 60 Minutes

television show

Despite such comments from patients, providers have expressed significant concerns about potential harm if patients access imaging reports before the provider has reviewed them; for example, in a study of 69 clinicians and 57 patients, 90% of the patients and 81% of the providers agreed that patient access to health information is necessary for delivery of high-quality care [15]. However, 73% of the physicians believed that immediate release would be more confusing than helpful, whereas 16% of the patients felt that it would be more helpful than confusing [15]. The study also found that 75% of the patients strongly felt that providers should contact them within 24 hours of the release of a result indicating a health concern, whereas only 9% of the clinicians agreed that communication should occur within this time frame [15].

### Report Format and Detail

Apart from the differences in perceived trust in physicians, preferences also vary regarding the amount of detail in the report and the format in which the results are presented. Short et al [16] surveyed 193 women to evaluate different ways of presenting breast imaging results. The authors found that compared to those receiving standard reports, participants who received a “web-based interactive report” spent more than twice as long viewing the report, had significantly higher self-reported comprehension scores, had significantly higher ratings of satisfaction with their interpreting radiologist, and significantly higher objective comprehension scores. These findings suggest that the format of communication of radiology reports may also influence the effective communication of results and patient satisfaction. This has been confirmed by results from other studies. A study analyzing physician perceptions of the use of electronic health records [17] reported several reactions from physicians about the role of computer skills:

Old generation doctors, whom I respect a lot of course, let’s say there is a urine culture results, they don’t know that there is a click where you can get the susceptibility.

Beyond technical barriers, physicians also emphasized the importance of maintaining interpersonal communication; for example, some physicians described verbally explaining their actions at the computer so that patients would not feel neglected:

Ok, now I am checking your results, I am checking your past file.

Despite differences in computer skills, most patients seem to be using modern technology regularly enough to be able to navigate patient portals. A recent study evaluated a commercially available software platform that delivered patient-centered radiology reports across all imaging modalities and found that more than half of the patients (59%) spent significantly more time (mean 5.8, SD 6.6 min vs mean 1.8, SD 1.9 min) using “interactive reports” than raw text reports [17]. The authors concluded that direct reporting with “interactive reports” improved understanding for the majority of patients (84.7%). However, it has been questioned whether this is true for all patients and their family members [18]. While clinicians seem to be protective of patient information from an ethical perspective, patients feel that it is their right to know their results before any conversations take place about follow-up care [19]:

I think it’s a good thing. I’d say thank you for doing it because it shouldn’t be a secret. I mean, people are people, and they’re [clinicians] not gods.

Apart from individual preferences, there is discussion about the ethical implications of direct reporting [18].

There is limited evidence to date on how to optimize these reports for patients, taking into account patient and provider preference heterogeneity. An international advocacy group that focuses on improving transparency between patients and providers has offered several perspectives on the implications of increasing patient access to clinic, laboratory, and radiology reports [20,21]. These studies have focused on the discrepancies between patient and clinician preferences regarding the sharing of health information. Providers expressed concern about engendering unnecessary anxiety among patients [4,22]. Nonetheless, few clinicians indicated that they would alter the language in their clinical notes, even knowing that patients would have access to them.

### Objectives

There remains a gap in knowledge concerning how the communication of imaging results contributes to patient and provider frustration and how communication preferences vary by provider characteristics. By better understanding preference heterogeneity among ordering providers and patients as well as how preferences depend on the level of detail of clinical reporting and the mode and timing of result communication, more nuanced and personalized imaging report communication methods could be designed.

The objective of this study was to compare and contrast patients and providers with respect to preferred source of imaging report information, preferred method of imaging report communication, and perceptions of patients’ understanding of imaging reports. We further analyzed provider preference heterogeneity regarding preferred first reporting method to identify the provider characteristics associated with communication preferences. We aim to leverage these results to explore options for a differentiated reporting approach that is more responsive to patient and provider needs.

## Methods

### Participants

Patients were recruited on site in the waiting rooms of a midsize academic medical center in the northeastern United States before their radiology appointment. QR codes linking to an online survey in REDCap (Research Electronic Data Capture; Vanderbilt University) were distributed [23,24], and a paper survey option was also available. Physical and digital flyers were displayed on tables and screens. The inclusion criteria were adult patients (aged ≥18 y) who had recently undergone x-ray, ultrasound, magnetic resonance imaging, or computed tomography procedures. Patients aged <18 years, non–English-speaking patients, and those who had never received radiology services were excluded.

Ordering providers were recruited by contacting department chairs via emails; announcements at departmental meetings and multidisciplinary conferences, including tumor boards; and by distributing physical flyers The inclusion criteria required respondents to be nonradiology ordering health care providers with radiology referral privileges from any nonradiology clinical department. Radiology health care providers were excluded because the focus of this study was on the preferences of ordering providers. The survey was completed in REDCap.

### Ethical Considerations

The institutional review board of the University of Vermont determined both the patient and provider surveys to be exempt from review. No consent process was necessary because the research posed no more than minimal risk of harm to participants and involved no procedures for which written consent is normally required outside the research context. Participants were not offered any compensation.

### Setting

The data were collected from providers at a center where patients can access most laboratory, radiology, and cardiology reports through a patient portal. Providers can add comments to help patients interpret these results. Reports are automatically released to the portal a specified number of days after they become available, with the delay depending on the type of result and its classification tier. Result batches are released hourly. Tier 1 laboratory tests, such as prothrombin time and cholesterol levels, are delivered to patients directly. Tier 2 laboratory tests, such as HIV or sexually transmitted disease tests, are more narrative or sensitive in nature and are deemed preferable to be given to the patient by a provider; hence, they are placed on delayed release to allow time for provider-patient communication. Certain results, such as secondary interpretations of imaging studies from other institutions, reports with comments about suspected abuse, and other flagged findings, are never released.

### Data

The survey was first validated through focus groups with patients. Patients were asked whether they had ever used the online portal to access their imaging results, whether they usually understood the content of the reports, and whom they consulted if they had questions about the reports. They were also asked when they typically wanted to know their imaging results, how they preferred to receive these results, and whether their preferences changed depending on the results being within or outside the reference range. The findings from these focus groups were then used to inform both patient and provider surveys.

The patient survey first asked whether participants were aware that patients can access their imaging results online, whether they had used the online portal before, and whom they would consult if they had questions about their results. It then asked where they usually obtained their results first when they were within the reference range and when they were outside the reference range and from whom they would prefer to learn their results when they were within the reference range and when they were outside the reference range. Additional questions addressed what patients believe a radiologist does and how much interaction they would like to have with their radiologist in the context of discussing imaging results. The survey also included questions about participants’ age, gender, race, income, educational level, usual source of care, source of imaging services, presence of chronic conditions, and health insurance status. Finally, participants were asked whether they were worried about directly obtaining their imaging results.

In the provider survey, ordering providers were asked how and how often they accessed their patients imaging results, their perceptions of how and when their patients accessed imaging results, and their preferences for how patients should access imaging results. The survey also included demographic questions on gender, race, marital status, medical degree and certification, specialty (medical or surgical), and number of years in practice.

### Statistical Methods

We used chi-square and *t* tests to determine whether there were significant differences between observed and expected frequencies. In addition to descriptive statistics, we used logistic regression model to predict the probability of MyChart (patient portal) being the preferred imaging reporting method based on individual patient and provider characteristics (yes or no). We analyzed which characteristics were predictors of preference for direct results reporting. Provider-level variables included gender, race, specialty, years of experience, perceived level of patient understanding of results, and preferred level of detail in reporting.

## Results

### Overview

In this study, we compared and contrasted patients and ordering providers with respect to preferred source of imaging report information, preferred method of imaging report communication, and perceptions of patients’ understanding of imaging reports. We also compared and contrasted ordering providers’ preferences for first method of imaging report communication. Overall, most providers reported that they believed that their patients had a low to moderate understanding of their imaging results and therefore should receive a less-detailed version of their reports. Ordering providers generally did not believe that results should be communicated by radiologists or radiologic technologists.

### Descriptive Data

**Figure 1:**
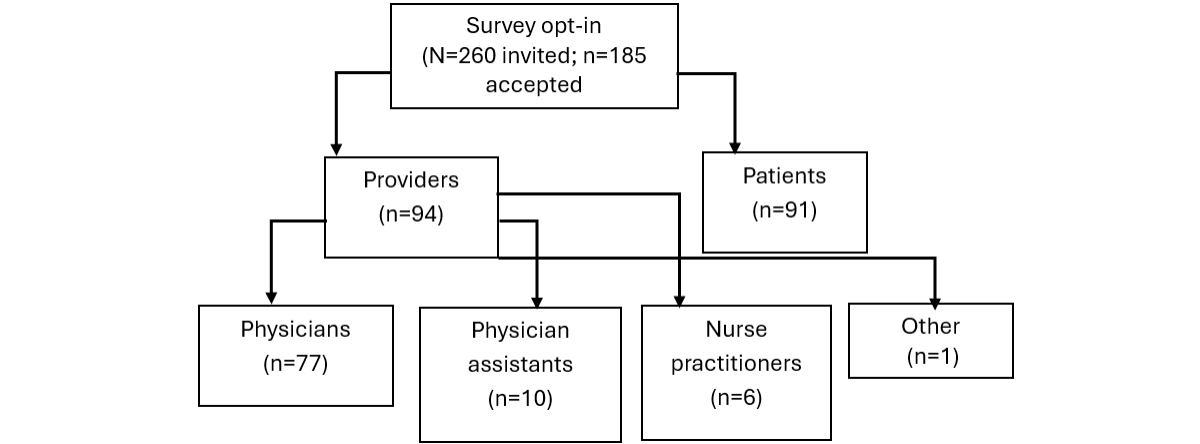
Flowchart depicting participant recruitment.

In total, 91 patients as well as 77 physicians, 10 physician assistants (PAs), 6 nurse practitioners (NPs) and 1 other physician completed the surveys. Figure 1 presents a flowchart of participant recruitment. Table 1 summarizes descriptive results, including gender, race and ethnicity, patients’ awareness of the online portal, providers’ perceptions of patients’ awareness and understanding of direct results, and both patients’ and providers’ preferences for information source and reporting method. We used chi-square and *t* tests to compare differences in group means and found significant differences between patients and providers for all measures.

**Table 1:** Descriptive statistics of patient and provider survey respondents.

Variables			Respondent groups, n (%)				
			Patients (n=91)		Providers (n=94)		Total (n=185)
**Gender**							
	Woman	70 (76.9)		53 (56.4)		123 (66.5)	
	Man	20 (22)		40 (42.6)		60 (32.4)	
	Nonbinary	1 (1.1)		0 (0)		1 (0.5)	
	Missing	0 (0)		1 (1.1)		1 (0.5)	
**Race and ethnicity**							
	Other races and ethnicities	17 (18.7)		9 (9.6)		26 (14.1)	
	White	73 (80.2)		80 (85.1)		153 (82.7)	
	Missing	1 (1.1)		5 (5.3)		6 (3.2)	
**Patient awareness of online portal**							
	Unaware	20 (22)		3 (3.2)		23 (12.4)	
	Aware, never used	28 (30.8)		5 (5.3)		33 (17.8)	
	Aware, have used	43 (47.3)		86 (91.5)		129 (69.7)	
**Perceptions of patients’ understanding of imaging reports**							
	Low or none	2 (2.2)		2 (2.1) (50)		4 (2.2)	
	Moderate	17 (18.7)		47 (50)		64 (34.6)	
	High	52 (57.1)		38 (40.4)		90 (48.6)	
	Don’t know	20 (22)		7 (7.4)		27 (14.6)	
**Preferred source of imaging report information**							
	Referring provider	78 (85.7)		84 (92.3)		162 (87.6)	
	Radiology staff	7 (7.7)		0 (0)		7 (3.8)	
	Friends or family	1 (1.1)		0 (0)		1 (0.5)	
	Internet	3 (3.3)		1 (1.1)		4 (2.2)	
	Other	2 (2.2)		9 (9.6)		11 (5.9)	
**Preferred method of imaging report communication**							
	Online portal	66 (72.5)		15 (16)		81 (43.8)	
	Telephone call from referring provider	19 (20.1)		23 (24.5)		42 (22.7)	
	Face-to-face meeting with referring provider	2 (2.2)		32 (34.0)		34 (18.4)	
	Telemedicine (Zoom) meeting with referring provider	0 (0)		8 (8.5)		8 (4.3)	
	Mailed copy of results	1 (1.1)		1 (1.1)		2 (1.1)	
	Other	3 (3.3)		15 (16.0)		18 (9.7)	

The majority of the patients (70/91, 77%) who completed the survey were women. Among the ordering providers, a little more than half (53/94, 56%) were women. Almost one-fifth of the patients (17/91, 19%) identified as members of racial and ethnic minoritized groups or preferred not to answer compared to 10% (9/94, 96%) of the providers were of racial and ethnic minorities. The ordering providers (86/94, 91%) overwhelmingly believed that patients were aware of direct reporting and had used the online portal. By contrast, less than half of the patients (43/91, 47%) said that they were aware of the portal and had used it. More than half of the ordering providers (49/94, 52%) believed that patient understanding of their imaging reports was low or moderate, whereas more 1 out of 5 patients (19/91, 21%) said that they had a either low or moderate understanding of their reports. More than half of patients believed they have high understanding of the reports (52/91, 57%).

There was more agreement on the source of imaging report information. A majority of both patients (78/91, 86%) and ordering providers (84/94, 89%) believed that the source of imaging report information should be the ordering provider. Some patients believed that it should be radiology staff (7/91, 8%), friends or family (1/91, 1%), or the internet (online; 3/91, 3%). Ordering providers who did not select themselves mostly chose “other” sources (11/94, 12%, and only 1% (1/94) chose the internet. A majority of the patients (66/91, 73%) believed that imaging report communication should occur through an online portal, whereas only 16% (15/94) of the ordering providers agreed. Instead, 33% (32/94) of the ordering providers preferred face-to-face communication, and 25% (23/94) preferred telephone communication.

When asked whether patients should receive a less-detailed version of their imaging reports, the ordering providers responded as follows: 7% (7/94) preferred “no access,” 46% (43/94) “a simplified patient version,” 20% (19/94) “summary only,” and 27% (25/94) “full report.” Two-thirds of the respondents (63/94, 67%) indicated that they would not want either a radiologist or radiologic technologist to communicate imaging results to patients. In addition, 85% (80/94) reported that patients should receive their imaging reports only after a delay to allow ordering providers to review the results. In summary, there was significant preference heterogeneity among the ordering providers regarding how they would want patients to access their imaging results.

Apart from preference heterogeneity between patients and providers, we analyzed heterogeneity among providers. Table 2 shows provider-specific experience and expertise. The majority of providers (77/94, 82%) held a Doctor of Medicine (MD); Doctor of Osteopathic Medicine (DO); or Bachelor of Medicine, Bachelor of Surgery degree. In addition, 6% (6/94) were NPs, and 11% (10/94) were PAs. On average, the providers had 15.6 (SD 10.0) years of experience. Ordering providers from a wide variety of specialties, including family medicine, orthopedic surgery, and pulmonology, participated in the study. Using logistic regression, we analyzed the differences in the preferred method of imaging report communication as a function of provider characteristics, including age, race, years of experience (>5 y), specialty (primary care vs other), degree (MD or DO vs NP or PA), as well as providers’ perceptions of patient understanding of reports (medium to high), whether they read the radiologist’s report (yes or no), and their satisfaction with reviewing imaging results with patients (high vs not high).

**Table 2:** Provider expertise and experience (n=94).

Variables			Values
**Degree or professional role, n (%)**			
	MD^a^, DO^b^, or MBBS^c^	77 (82)	
	Nurse practitioner	6 (6)	
	Physician assistant	10 (11)	
	Other	1 (1)	
Experience (y), mean (SD)			15.6 (10)
**Specialty, n (%)**			
	Cardiology	1 (1)	
	Endocrinology	2 (2)	
	Family medicine	22 (24)	
	Gastroenterology	1 (1.1)	
	Internal medicine	1 (1)	
	Obstetrics and gynecology	6 (6)	
	Oncology (medical)	4 (4)	
	Oncology (radiation)	2 (2)	
	Orthopedic surgery	10 (11)	
	Pulmonology	11 (12)	
	Surgery (general)	6 (6)	
	Surgery (colorectal)	3 (3)	
	Surgery (thoracic)	1 (1)	
	Surgery (vascular)	1 (1)	
	Urology	5 (5)	
	Other	16 (17)	

^a^MD: Doctor of Medicine.

^b^DO: Doctor of Osteopathic Medicine.

^c^MBBS: Bachelor of Medicine, Bachelor of Surgery.

### Statistical Analysis

Table 3 shows the probability that providers prefer MyChart as their preferred method of imaging report communication (yes or no). All coefficients had the expected signs, based on our prior hypothesis about the factors affecting a preference for online reporting. Female providers were more likely to choose MyChart as their preferred results reporting method (OR 0.0667; *P*=.05), and White providers were less likely to prefer MyChart than providers from racial and ethnic minoritized groups (marginal effect −0.2786, SE −0.2786; *P*=.005). Primary care providers were 34.1% more likely to prefer MyChart over other reporting methods, such as telephone, face-to-face, or telemedicine (*P*=.002). Those who held MD, DO, or Bachelor of Medicine, Bachelor of Surgery degrees were 20.8% less likely to prefer communication via MyChart than NPs or PAs (*P*=.02).

**Table 3:** Logistic regression results, marginal effects, and odds ratios.

Variables	MyChart: preferred method of imaging report communication	
	Marginal effect (SE)	Odds ratio (95% CI)
Female	0.1278^a^ (0.0652)	0.0678^a^ (−0.9870 to 2.2930)
White	−0.2786^b^ (−0.0268)	0.0561^b^ (−0.9867 to 2.2930)
Primary care provider (yes—vs specialty care)	0.3412^b^ (0.1077)	1.7933^b^ (0.0128 to 7.7918)
MD^c^ or DO^d^ (yes—vs nurse practitioner or physician assistant)	−0.2077^a^ (0.0860)	0.0431^a^ (−0.7865 to 1.1872)
>5 y of experience (yes)	−0.0581 (0.1024)	0.0187 (0.2215 to 0.0388)
Patients have a medium or high understanding (yes)	0.1944^b^ (0.0625)	1.9726^b^ (−0.8088 to 2.2741)
Read the radiologist’s report	−0.0625 (0.0951)	0.0391 (0.0168 to 0.6742)
Highly satisfied with process of reviewing results with patients	−0.2930^b^ (0.1165)	0.14122^b^ (−2.9717 to 0.9454)
Desired report detail level—full report	0.1768^b^ (0.0502)	0.0731^b^ (0.0139 to 0.1635)

^a^*P*<.05.

^b^*P*<.01.

^c^MD: Doctor of Medicine.

^d^DO: Doctor of Osteopathic Medicine.

Having >5 years of experience did not significantly affect the probability of preferring MyChart over other reporting methods (marginal effect −0.0581, SE 0.1024; *P*=.57). Providers who believed that patients had a medium or high understanding of their imaging results were 19.4% more likely to prefer MyChart (*P*=.002). Whether the ordering provider wanted a radiologist to review the imaging results before sharing them with patients did not significantly affect the probability of preferring MyChart as the reporting method (marginal effect −0.0625, SE 0.0951; *P*=.51). Ordering providers who reported high satisfaction with reviewing imaging results with patients directly were 29.3% less likely to prefer MyChart as the reporting method (*P*=.01). Providers who preferred that patients have access to the full report of imaging results were 17.7% more likely to prefer MyChart as the first reporting method (*P*<.001).

## Discussion

### Principal Findings

This study identified three main drivers of the well-documented dissatisfaction with the current process of communicating imaging results to patients. First, although patients and most providers believed that patients should have access to their imaging reports, ordering providers generally preferred that report release be delayed to allow for direct communication. Second, in general, patients believed that they have a better understanding of reports than ordering providers think they do. Third, dissatisfaction was driven by the lack of flexibility in the current mandated communication methods, which do not accommodate providers’ preference heterogeneity regarding the communication of imaging results to patients. These findings suggest that, similar to patients, providers have diverse preferences for how results should be communicated to patients. Therefore, the most preferred communication method should be determined collaboratively, taking into account the preferences of both the provider and the patient.

The results of this study have several implications. First, they suggest that shared decision-making between patients and providers could help establish which first method of communication is preferred in a given situation. Shared or patient-centered decision-making—the process by which a health care provider communicates to the patient personalized information about options, outcomes, probabilities, and uncertainties regarding the available options; and the patient communicates values and the relative importance of benefits and harms [25]—is important for providing care consistent with patient preferences and may improve satisfaction and adherence [26]. Allowing providers more time to better understand patients’ preferences for direct results communication could enable differentiation of direct reporting based on patients’ needs. The context may matter as well: complex illnesses require weighing the risks and benefits of different treatments, and patients with multiple illnesses may similarly require careful consideration [27]. Shared decision-making should occur before any direct communication of results through online portals.

Second, interactive radiology reports may improve communication effectiveness by including images; graphs; tables; and interactive elements such as hyperlinks to annotated, scrollable images and additional resources [28]. Again, it would be key to differentiate reporting methods based on patient preference heterogeneity. To better understand patients’ individual preferences, further research is needed, along with direct patient-provider communication. Patients may be interested in hearing their provider’s perspective on how direct reporting of results should be conducted. Generally, the results of this study indicate a strong provider preference for delaying the release of imaging reports to patients, which is supported by previous research. This issue has been addressed in the literature; for instance, a survey of 48 ordering physicians by Henshaw et al [29] demonstrated that as the complexity of a radiology report increases (x-rays vs computed tomography or magnetic resonance imaging), providers believe that there should be a longer embargo period to account for this added complexity. Leonard et al [15] also found that 62.7% of physicians believed that immediate release would be more confusing than helpful, whereas 15.8% of patients felt that it would be more helpful than confusing; in addition, 75.1% of patients strongly felt that providers should contact them within 24 hours of the release of a result that indicated a health concern, whereas only 9% of clinicians agreed with this time frame.

Third, the introduction of the Cures Act occurred without careful consideration of the practical workflow constraints faced by providers, such as time pressures, workload, and system limitations, that may shape their communication preferences. In addition, variation in health literacy might impact patient understanding, suggesting a need to tailor communication approaches accordingly. However, such approaches involve practical challenges, including the costs of building individual customized communication systems and ensuring protection against cyberattacks, which remain a common and costly concern [30,31].

Future research should focus on exploring how the complexity of imaging examinations could influence both patient and provider communication preferences. Such work should also analyze differences across service lines and provider specialties. Our survey results indicated differences between primary care ordering providers and specialty providers, consistent with prior literature; for example, Donohue [32] reviewed studies on the use of health care resources and the quality of care provided by generalist versus specialist providers and found that while care is often equivalent for the management of common conditions such as diabetes or hypertension, specialists tend to outperform generalists in their areas of expertise. However, there is wide intergroup variability with However, substantial intergroup variability exists, with greater heterogeneity of care observed within both generalist and specialist groups. Our findings suggest that it is an oversimplification to assume that all providers share similar preferences regarding the communication of imaging results to patients.

Once qualitative data or discrete choice analyses have established the nature of preference heterogeneity among patients and providers, more concrete and actionable recommendations could be developed for health systems on implementing differentiated reporting strategies and shared decision-making processes, considering policy and technology factors.

### Limitations

This study has several limitations. Participants in both the patient and provider surveys were self-selected, potentially introducing selection bias. The study population was not representative of a larger, national population of patients and providers. In addition, the vast majority of the patient survey participants (87/91, 96%) completed the online form using a QR code, which may underrepresent individuals with limited access to or familiarity with technology. Due to these potential sources of selection bias—self-selection and technological access requirements—the findings cannot be generalized. The sample sizes were also too small for detailed subgroup analyses. Nevertheless, the study results underscore the need for additional research to inform policy development, guide clinical practice, and improve the rollout of the Cures Act.

Our findings also indicate differing perceptions of the best ways for patients to interact with their providers regarding imaging reports. Generally, health literacy plays an important role in discussions of direct results reporting, which the Cures Act does not sufficiently consider. The complexity of imaging studies might influence patient understanding and communication preferences. Additional research is needed to better understand the effect of health literacy on patient understanding and interpretation of results to design effective public health interventions aimed at improving the gap between patient understanding and direct reporting.

### Conclusions

There is a paucity of guidance from national societies, including the American College of Radiology, American College of Pathology, and American Medical Association, on this topic. Under the Health Insurance Portability and Accountability Act privacy rule, patients are guaranteed complete access to their medical image records, with limited exceptions. While there are privacy concerns surrounding the cybersecurity of patient portals, the rapid expansion of electronic health system capabilities and the growing emphasis on patient empowerment underscore the need for understanding how patients and providers perceive patients’ access to their medical records. This study illustrates that there is room for a more nuanced and differentiated approach to reporting methods, in which patients may choose to access their results directly while also having other options available.

The results suggest that shared decision-making between patients and providers should occur before the reporting of results to establish patient and provider expectations and help facilitate efficient and effective health communication. Improved communication should also clarify who provides the imaging reports (eg, radiologists vs radiologic technologists), the levels of access patients and providers have to these reports, and whether reporting practices differ between primary care and specialty providers. In addition, clearer expectations are needed regarding how quickly reports will be available in clinicians’ offices, as this may explain variations in preferences about the timing of patient access.

## Abbreviations

DO: Doctor of Osteopathic Medicine
GP: general practitioner
MD: Doctor of Medicine
NP: nurse practitioner
PA: physician assistant
REDCap: Research Electronic Data Capture
